# Supplementary material for: A molecular staging model for accurately dating the endometrial biopsy
Source: Nat Commun. 2023 Oct 6;14:6222. doi: 10.1038/s41467-023-41979-z (PMC10556104; doi:10.1038/s41467-023-41979-z)
Supplement: Supplementary file 1 — Supplementary Information [file 41467_2023_41979_MOESM1_ESM.pdf]

Supplementary Figure 1. Validation model using PCA and k-means clustering. The PCA plot from the unsupervised validation model coloured by k-means assigned clusters. The clusters starting from 1 and moving anticlockwise (3, 5, 2, 4) were reordered and used as a proxy for cycle time to construct the unsupervised model.

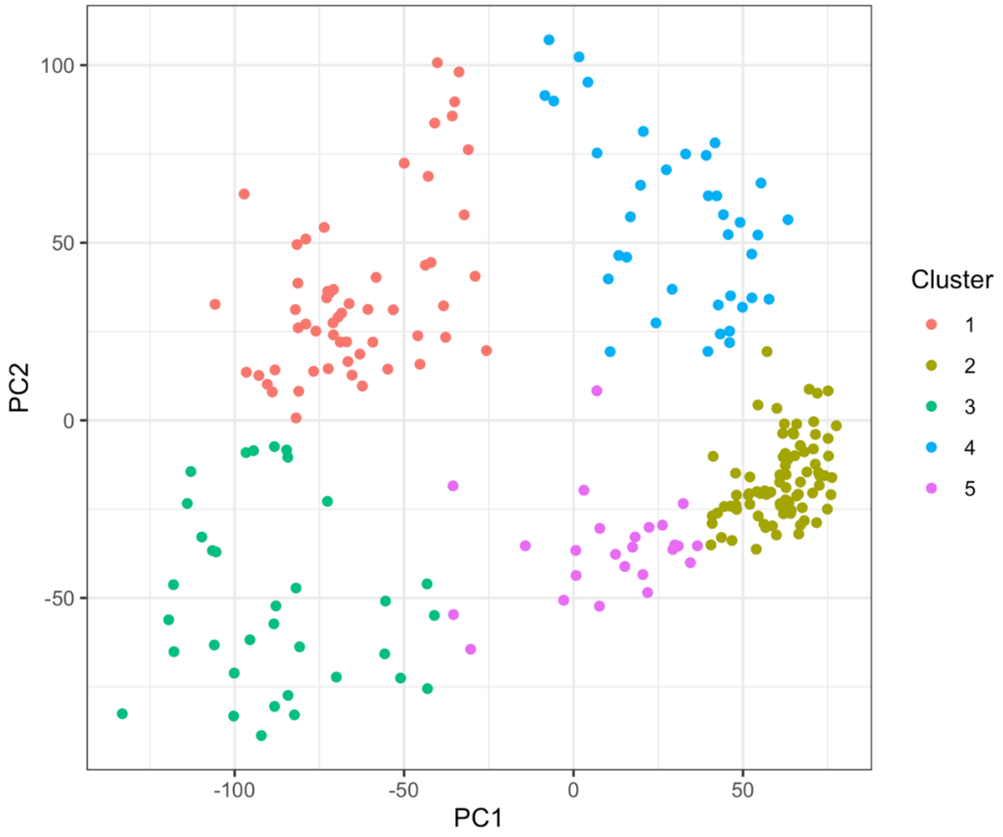

Supplementary Figure 2. Performance of the unsupervised validation model. Model time estimates from the validation model were compared to the molecular staging model. Since time 0 in the validation model is not expected to correspond to the beginning of menstruation, the time is offset using the time of the earliest menstrual sample identified by the molecular staging model.

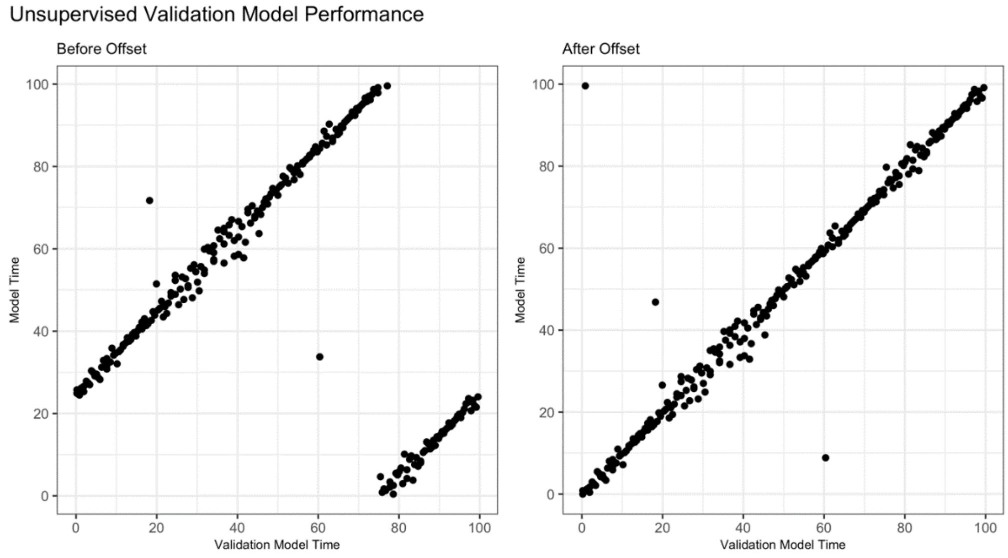

Supplementary Figure 3. RNA-seq data from GSE180485 [1] with the left hand plot showing samples labelled with our Model cycle time as a % of the way through the cycle, and the right hand plot using the Lipecki et al model EndoTime (eLH+) estimates. Note that molecular model % values show a steady progression through the cycle in concordance with the PCA plot. Conversely, the eLH+ day 4-5 data points are widely spread (i.e. these samples have different molecular profiles), and some of the eLH+ days 8-10 samples are grouped together showing that EndoTime cannot reliably discriminate between samples from these post LH surge days.

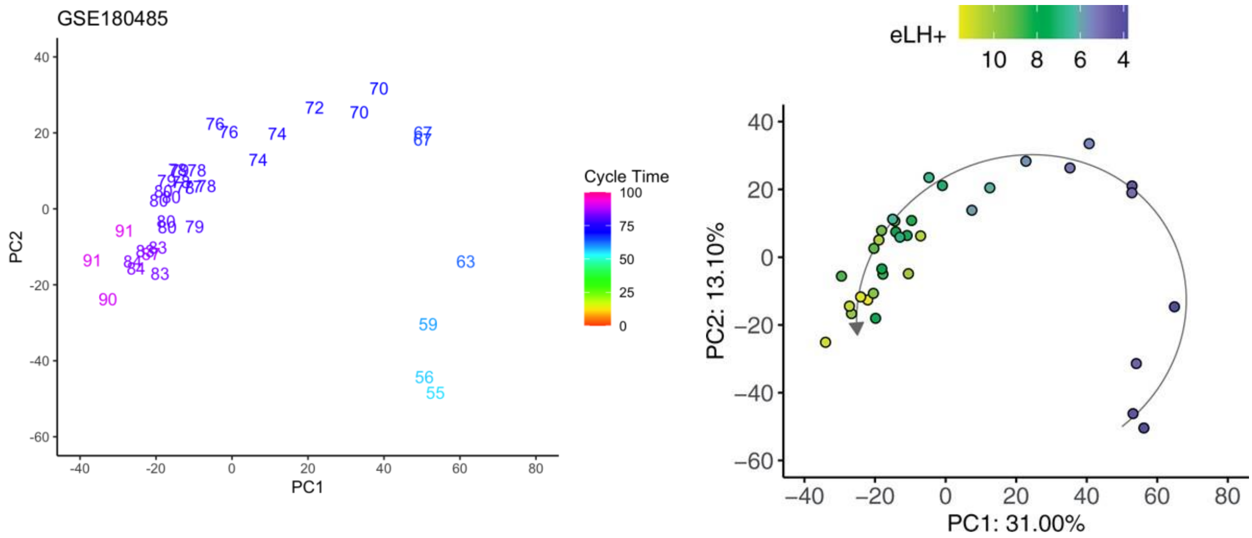

Supplementary Figure 4. The impact of ancestry on endometrial gene expression. Examples of genes showing significantly different endometrial expression between women of different ancestries. Lists of differentially expressed genes for ancestry are in Supplementary Data 3. Ancestry information was obtained from a previously published study [2]. (EAS East Asian, SAS South Asian, EUR European).

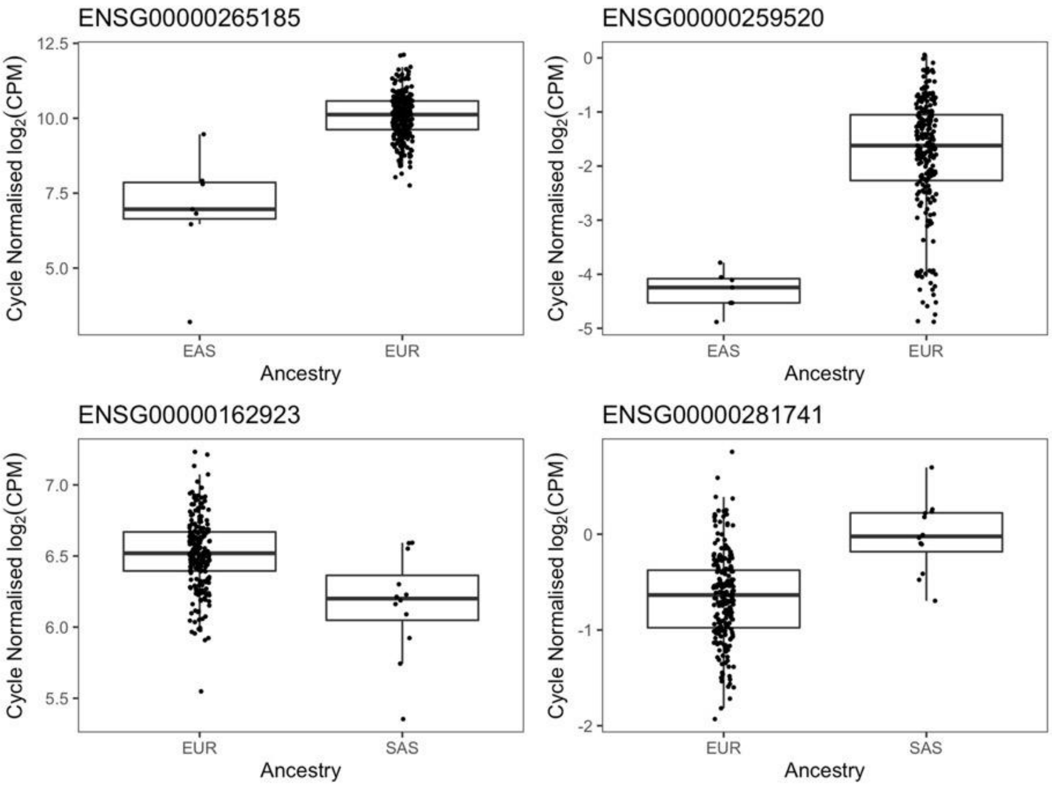

Supplementary Figure 5. Of the 238 genes listed in the original endometrial receptivity assay (ERA, [3] https://pubmed.ncbi.nlm.nih.gov/20619403/), 207 were recognised in our NGS data, and 70% of these (145/207) changed expression significantly between cycle times 66±2 and 76±2 (POD 3-7). This figure shows the 6 most significantly down-regulated genes and the 6 most significantly up-regulated genes from among the 145 significant ERA genes that we identified.

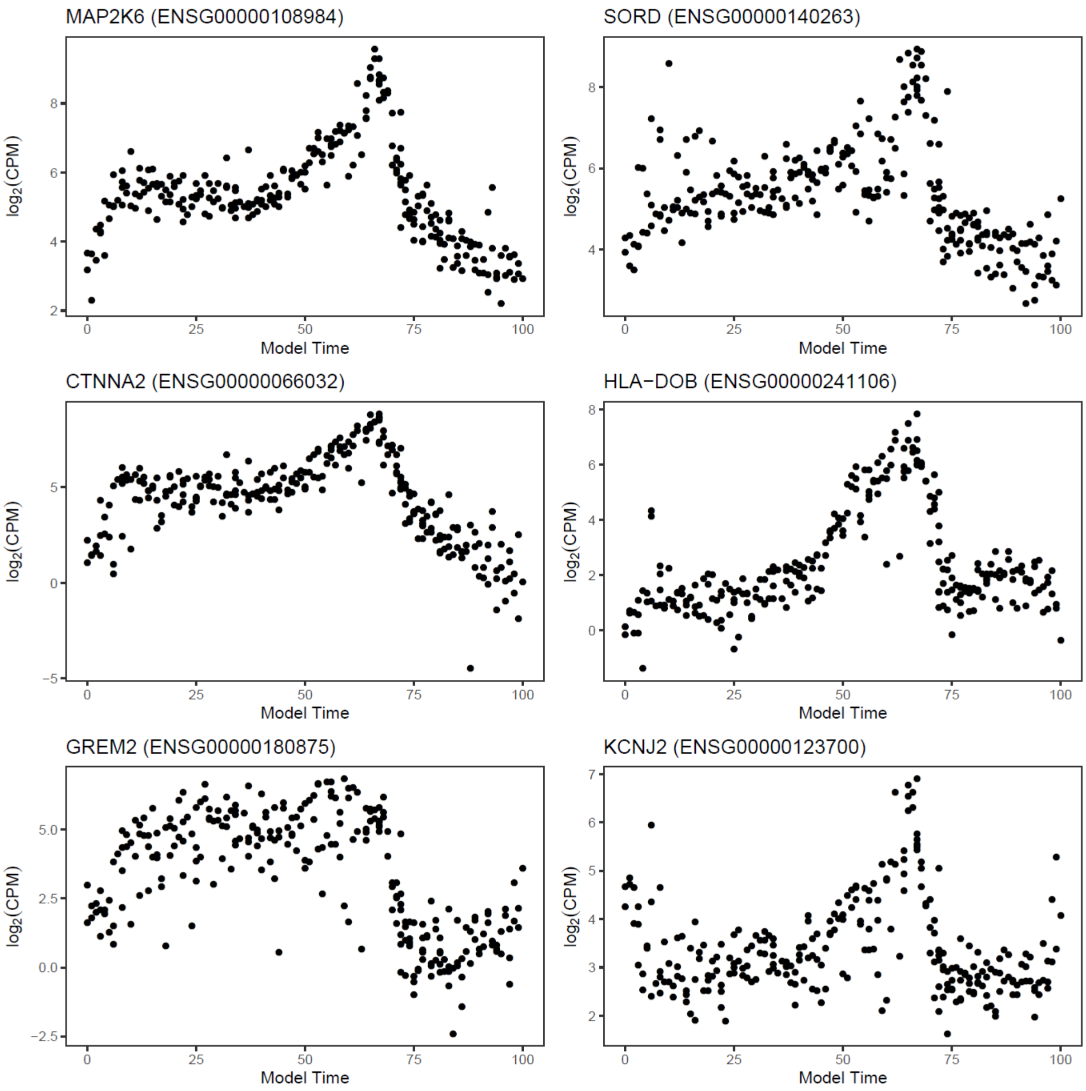

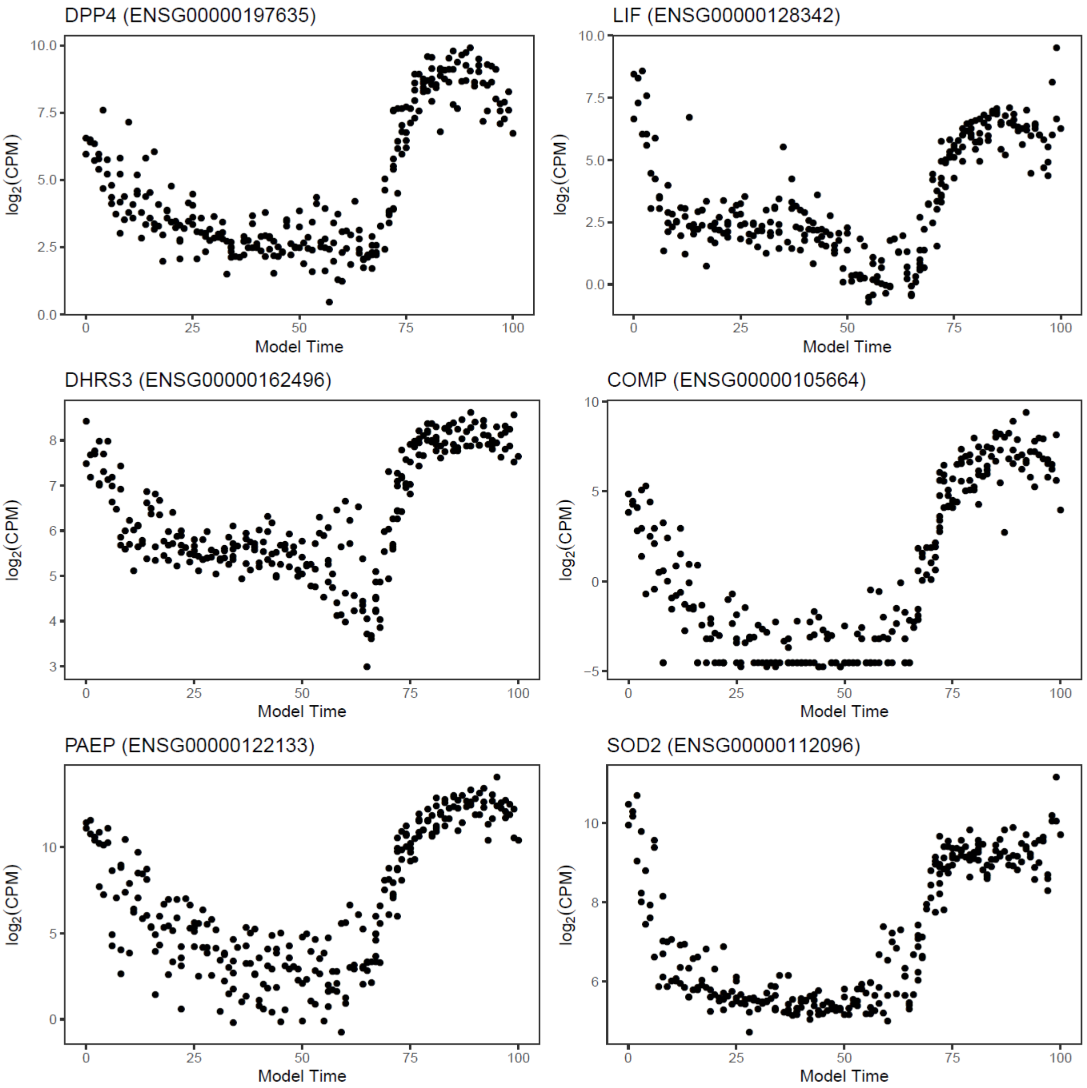
DPP4 (ENSG00000197635)
LIF (ENSG00000128342)
DHRS3 (ENSG00000162496)
COMP (ENSG00000105664)
PAEP (ENSG00000122133)
SOD2 (ENSG00000112096)
log2(CPM)
Model Time
0
25
50
75
100

Supplementary Table 1. Pregnancy outcomes for 236 women who provided endometrial biopsies from which the final molecular staging model was developed. Note 25% of the cohort had had a successful pregnancy and only 3% reported a miscarriage.

Pregnancy outcomes	Number of women (% of total population)	Age in years at time of endometrial biopsy (average and range)	Previous pregnancies at time of endometrial biopsy (average and range)	Live births at the time of endometrial biopsy (average and range)
Pregnant with miscarriage	6 (3%)	30.3 (21-38)	1.7 (1-3)	0.0
Pregnant with termination	15 (6%)	30.5 (23-38)	1.3 (1-3)	0.0
Pregnant with no live birth, reason not given	15 (6%)	32.2 (19-42)	1.8 (1-4)	0.0
Pregnant with at least 1 live birth	60 (25%)	35.4 (22-48)	2.4 (1-6)	1.7 (1-4)
Never pregnant	140 (59%)	29.2 (18-49)	0.0	0.0
TOTAL	236	31.1 (18-49)		

Supplementary Table 2. Fertility intentions for 236 women who provided endometrial biopsies from which the final molecular staging model was developed. Note only 28 women reported problems conceiving, of whom 10 went on to have successful pregnancies.

Fertility intentions	Group total N= (% of total cohort)	Never pregnant N= (% of group total)	Endometriosis N= (% of never pregnant)	Gravida N= (% of group total)	Endometriosis N= (% of Gravida)	Parity N= (% of group total)	Endometriosis N= (% of Parity)
Never tried to get pregnant	68 (29%)	53 (78%)	33 (62%)	15 (22%)	12 (80%)	2 (3%)	1 (50%)
Never tried for more than 12 months to get pregnant	40 (17%)	9 (23%)	8 (89%)	31 (78%)	19(61%)	25 (63%)	14 (56%)
Tried for more than 12 months to get pregnant	28 (12%)	14 (50%)	12 (86%)	14 (50%)	9 (64%)	10 (36%)	6 (60%)
Did not provide information on fertility intentions	100 (42%)	64 (64%)	50 (78%)	36 (36%)	25 (69%)	23 (23%)	11 (48%)
TOTALS	236 (100%)	140 (59%)	103 (74%)	96 (41%)	65 (68%)	60 (25%)	32 (53%)

Supplementary Table 3. Numbers of endometrial genes that change expression significantly with age. Results are from 12,808 genes in common between our data set and GSE141549. Sub analysis by cycle stage shows that the majority of the genes that showed significant changes with age were found in samples taken in the secretory phase. NGS_age = RNA-seq samples from the current study. GSE_age = samples from GSE141549.

	NGS_age N=266	GSE_age N=87	combined_age N=353
Combined (all samples)	32	65	206
Menstrual only	0	0	0
Proliferative only	0	0	4
Secretory only	163	0	218

Supplementary Table 4. Numbers of endometrial genes that show significant differences in expression between subjects of differing ancestries. Statistical analysis used two-sided empirical Bayes moderated t-tests implemented in limma [4] with corrections for multiple comparisons performed using the Benjamini-Hochberg method. AFR = African, AMR = American, EAS = East Asian, EUR = European, SAS = South Asian. Ancestry information was obtained from a previously study [2]. Gene lists can be found in Supplementary Data 3.

**ANCESTRY**	N= (%)	AFR	AMR	EAS	EUR	SAS
Admixed	8 (3.1%)	3	1	2	15	-
AFR	2 (0.8%)		1	7	8	7
AMR	2 (0.8%)			-	-	-
EAS	7 (2.7%)				4	1
EUR	231 (88.2%)					4
SAS	12 (4.6%)					

REFERENCES FOR SUPPLEMENTARY MATERIAL

1. Lipecki, J., et al., *EndoTime: non-categorical timing estimates for luteal endometrium.* Hum Reprod, 2022. **37**(4): p. 747-761.
2. Mortlock, S., et al., *Tissue specific regulation of transcription in endometrium and association with disease.* Hum Reprod, 2020. **35**(2): p. 377-393.
3. Diaz-Gimeno, P., et al., *A genomic diagnostic tool for human endometrial receptivity based on the transcriptomic signature.* Fertil Steril, 2011. **95**(1): p. 50-60, 60 e1-15.
4. Ritchie, M.E., et al., *limma powers differential expression analyses for RNA-sequencing and microarray studies.* Nucleic Acids Res, 2015. **43**(7): p. e47.
