# Supplementary material for: A molecular staging model for accurately dating the endometrial biopsy
Source: Nat Commun. 2023 Oct 6;14:6222. doi: 10.1038/s41467-023-41979-z (PMC10556104; doi:10.1038/s41467-023-41979-z)
Supplement: Supplementary file 3 — Description of Additional Supplementary Files [file 41467_2023_41979_MOESM3_ESM.pdf]

Description of Additional Supplementary Files

Title: Supplementary Data 1

Description: Endometrial genes that show significant differential expression with increasing age of subject. Statistical analysis included n=353 subjects and used two-sided empirical Bayes moderated t-tests implemented in limma with corrections for multiple comparisons performed using the Benjamini-Hochberg method. Summary data of samples are presented in Supplementary Table 3.

Title: Supplementary Data 2

Description: Biological pathways significantly up and down regulated in secretory phase human endometrium (molecular staging model 58-100%) with increasing age. Statistical analysis used hypergeometric tests as implemented in clusterProfiler. Corrections for multiple comparisons were performed using the Benjamini-Hochberg method.

Title: Supplementary Data 3

Description: Endometrial genes that show significant differences in expression between subjects of differing ancestries. Statistical analysis used two-sided empirical Bayes moderated t-tests implemented in limma with corrections for multiple comparisons performed using the Benjamini-Hochberg method. AFR = African, AMR = American, EAS = East Asian, EUR = European, SAS = South Asian.
