# Supplementary material for: A molecular staging model for accurately dating the endometrial biopsy
Source: Nat Commun. 2023 Oct 6;14:6222. doi: 10.1038/s41467-023-41979-z (PMC10556104; doi:10.1038/s41467-023-41979-z)
Supplement: Supplementary file 7 — Reporting Summary [file 41467_2023_41979_MOESM7_ESM.pdf]

nature portfolio

Corresponding author(s): Peter AW Rogers
Last updated by author(s): Sep 20, 2023

Reporting Summary

Nature Portfolio wishes to improve the reproducibility of the work that we publish. This form provides structure for consistency and transparency in reporting. For further information on Nature Portfolio policies, see our Editorial Policies and the Editorial Policy Checklist.

Statistics

For all statistical analyses, confirm that the following items are present in the figure legend, table legend, main text, or Methods section.

n/a	Confirmed	
[ ]	[x]	The exact sample size (*n*) for each experimental group/condition, given as a discrete number and unit of measurement
[x]	[ ]	A statement on whether measurements were taken from distinct samples or whether the same sample was measured repeatedly
[ ]	[x]	The statistical test(s) used AND whether they are one- or two-sided<br>*Only common tests should be described solely by name; describe more complex techniques in the Methods section.*
[ ]	[x]	A description of all covariates tested
[ ]	[x]	A description of any assumptions or corrections, such as tests of normality and adjustment for multiple comparisons
[ ]	[x]	A full description of the statistical parameters including central tendency (e.g. means) or other basic estimates (e.g. regression coefficient) AND variation (e.g. standard deviation) or associated estimates of uncertainty (e.g. confidence intervals)
[ ]	[x]	For null hypothesis testing, the test statistic (e.g. *F*, *t*, *r*) with confidence intervals, effect sizes, degrees of freedom and *P* value noted<br>*Give P values as exact values whenever suitable.*
[x]	[ ]	For Bayesian analysis, information on the choice of priors and Markov chain Monte Carlo settings
[x]	[ ]	For hierarchical and complex designs, identification of the appropriate level for tests and full reporting of outcomes
[x]	[ ]	Estimates of effect sizes (e.g. Cohen's *d*, Pearson's *r*), indicating how they were calculated

Our web collection on statistics for biologists contains articles on many of the points above.

Software and code

Policy information about availability of computer code

Data collection	Clinical data were collected and stored using the REDCap database.
Data analysis	QC, alignment, quantification: FastQC v0.11.7; Trimmomatic v0.36; HISAT2 v2.0.5; StringTie v1.3.1<br>Downstream RNA-seq analysis: R v3.6.1; sva v3.32.1; limma v3.40.6; edgeR v3.26.8; mgcv vl.8-28; clusterProfiler v3.12.0; ReactomePA v.28.0<br>Downstream microarray analysis: lumi v2.36.0<br>Code Availability: The code for our model is available as an R package at https://github.com/jessicachung/endest (DOI: 10.5281/zenodo.8321573) and an R Shiny application is available at https://github.com/jessicachung/endspect. Analysis scripts for this manuscript can be found at https://github.com/jessicachung/endo_model_paper. Endest is published pursuant to the terms located in the applicable repository at github.com which terms permit reproduction,p ublication and adaptation of endest solely for non-commercial purposes. Publication of endest at github.com is not subject to the publication terms applicable to Nature publications.

For manuscripts utilizing custom algorithms or software that are central to the research but not yet described in published literature, software must be made available to editors and reviewers. We strongly encourage code deposition in a community repository (e.g. GitHub). See the Nature Portfolio guidelines for submitting code & software for further information.

Data

Policy information about availability of data

All manuscripts must include a data availability statement. This statement should provide the following information, where applicable:

- Accession codes, unique identifiers, or web links for publicly available datasets
- A description of any restrictions on data availability
- For clinical datasets or third party data, please ensure that the statement adheres to our policy

RNA-seq data has been deposited in the Gene Expression Omnibus database under accession number GSE234354 and the Illumina HT-12 microarray data under accession number GSE234368. Additional public datasets used are available on GEO with accession numbers GSE65099 (PMID: 26418742), GSE141549 (PMID: 32859947) and GSE180485 (PMID: 35092277). Homo sapiens reference genome GRCh38 and genome annotation were obtained from Ensembl (release 91). Source data are provided with this paper.

Human research participants

Policy information about studies involving human research participants and Sex and Gender in Research.

Reporting on sex and gender	This endometrial transcriptomics study by definition only recruited people born with a uterus. All subjects were reproductive age women with intact uteri that were experiencing normal menstrual cycles.
Population characteristics	The median age of subjects at time of endometrial biopsy was 33 years (range 18-49). Of the total of 358 subjects, 214 had confirmed endometriosis, 131 did not have endometriosis and in 13 endometriosis status was unknown. Similarly, 167 had had a prior pregnancy, 183 had never been pregnant, and pregnancy status information was unavailable for the remaining 8. Subjects undergoing laparoscopy for suspected endometriosis (Study 1) nearly all reported some degree of pelvic pain, and subjects from the IVF program (Study 2) had primary or secondary infertility. Detailed clinical data on other gynaecological conditions were not routinely collected, and all subjects reported regular menstrual cycles. Ancestry was not a selection criteria and was typical for patients seen at a public hospital in Melbourne. Of those for whom ancestry data were available, N=2 were African, N=2 American, EN=7 East Asian, N-231 European, N=12 South Asian and N=8 Admixed.
Recruitment	A total of 358 endometrial samples were collected for this study, comprising 264 samples taken from women at the time of surgery for suspected endometriosis ('Study 1') and 94 samples from individuals undergoing IVF ('Study 2'). All subjects were premenopausal and free from hormone treatment at the time of biopsy. Subjects were approached by either a member of the clinical team or a research nurse and provided with information about the study. If consent was given subjects were recruited. Subjects were free to withdraw at any time. All subjects recruited between 2012 and 2018 who met the selection criteria were included in the study. Of the 236 women whose endometrial data were used to develop the final molecular model, 173 were diagnosed with endometriosis. 60 of these 236 women had delivered 1 or more live births, with 38 of these 60 also being diagnosed with endometriosis, which suggests that if there are differences in the endometrium of women with endometriosis, they are either not universal, or not severe enough to preclude normal endometrial function. Our data suggests that the predominant driver of changing endometrial gene expression is menstrual cycle stage, and not phenotypic or pathological factors.
Ethics oversight	Studies were approved by the Human Research Ethics Committee of the Royal Women's Hospital, Melbourne, Australia (Projects 11-24 and 16-43 for Study 1) and Melbourne IVF (Project 13/17 for Study 2), and all subjects gave written informed consent.

Note that full information on the approval of the study protocol must also be provided in the manuscript.

Field-specific reporting

Please select the one below that is the best fit for your research. If you are not sure, read the appropriate sections before making your selection.

☒ Life sciences ☐ Behavioural & social sciences ☐ Ecological, evolutionary & environmental sciences

For a reference copy of the document with all sections, see nature.com/documents/nr-reporting-summary-flat.pdf

Life sciences study design

All studies must disclose on these points even when the disclosure is negative.

Sample size	Due to the nature of the study, no sample-size calculations were performed. To develop the full molecular staging model, we collected endometrial biopsies from women at the time of surgery for suspected endometriosis (n=236). An additional 19 samples collected from an IVF fertility study were used to develop the secretory molecular staging model (n=96 total). Based on previous studies examining the endometrial cycle stage and finding significant biological changes with smaller sample sizes (e.g. PMID: 15501903; PMID: 16306079) our sample size was expected to be sufficient. Additionally, our histopathology dating showed sufficient sample coverage across all endometrial cycle stages.
Data exclusions	To develop the molecular staging models, samples that could not be accurately dated (e.g. insufficient tissue, asynchronous glands) were

	excluded. Patients that were on progestin were also excluded. Additionally, for the secretory molecular cycle staging model, we excluded samples where disagreement between two independent pathology reports was greater than 2 days.
Replication	Illumina HT-12 microarray data (n=198) was used to build an independent validation model. Model cycle time was also checked against peripheral blood progesterone (n=187) and estrogen (n=159). Additionally, Illumina microarray data from GSE141549 and RNA-seq data from GSE65099 was used to demonstrate the model time from the molecular staging model was consistent with PCA plots.
Randomization	Randomisation for menstrual cycle stage was not done due to the nature of the study. It was assumed that patients presenting for surgery in a public hospital system where bookings are made many months in advance approximated a uniform distribution across the menstrual cycle. Hence it was assumed that the 236 endometrial samples used to develop the staging model were approximately uniformly distributed across the cycle, and the data was transformed so that the distance in time between each sample was identical. This assumption was supported by histopathology dating which demonstrated good sample coverage across all cycle stages.
Blinding	Blinding was not done due to the nature of the study. To develop the full cycle stage model, endometrial samples from all 236 subjects across all cycle stages were utilised. There were no treatment or control subgroups to allow blinding to occur.

Reporting for specific materials, systems and methods

We require information from authors about some types of materials, experimental systems and methods used in many studies. Here, indicate whether each material, system or method listed is relevant to your study. If you are not sure if a list item applies to your research, read the appropriate section before selecting a response.

Materials & experimental systems

n/a	Involved in the study	
☒	☐	Antibodies
☒	☐	Eukaryotic cell lines
☒	☐	Palaeontology and archaeology
☒	☐	Animals and other organisms
☐	☒	Clinical data
☒	☐	Dual use research of concern

Methods

n/a	Involved in the study	
☒	☐	ChIP-seq
☒	☐	Flow cytometry
☒	☐	MRI-based neuroimaging

Clinical data

Policy information about clinical studies
All manuscripts should comply with the ICMJEguidelines for publication of clinical research and a completedCONSORT checklist must be included with all submissions.

Clinical trial registration	This study was not a clinical trial. Limited clinical data were collected (age, endometrial histopathology, reason for surgery (endometriosis or infertility) but this information was used to develop a model for determining menstrual cycle stage for the whole cohort based on endometrial transcriptomics. There were no control or treatment groups, and the majority of the transcriptomic data had already been published as part of other studies: Some of the 358 subjects have had data published as part of previous studies investigating genetic regulation of endometrial gene transcription (Fung, Girling et al. 2017) (N=123 Illumina Human HT-12 v4.0 samples), (Fung, Mortlock et al. 2018) (N=229 Illumina Human HT-12 v4.0 samples), (Holdsworth-Carson, Chung et al. 2020, Mortlock, Kendarsari et al. 2020) (N=169 & 206 RNA sequencing (RNA-seq) samples respectively).
Study protocol	This was not a clinical trial and hence there wasn't a clinical trial study protocol.
Data collection	Endometrial samples were collected between 2012 and 2018 at the Royal Women's Hospital in Melbourne from patients undergoing surgery for either endometriosis or infertility. Samples were shipped to University of Queensland for Illumina HT-12 Microarray and RNA-seq analysis.
Outcomes	The primary outcome of this study was to develop and validate a a novel molecular staging model for accurately dating the endometrial biopsy. Secondary outcomes were to use the new model to investigate the effects of age and ancestry on the endometrial transcriptome.
